# Outcomes of prostate artery embolisation for benign prostatic hyperplasia in 10 cases at Steve Biko Academic Hospital

**DOI:** 10.4102/sajr.v23i1.1349

**Published:** 2019-02-11

**Authors:** Hatty G. Fischer, Farhana E. Suleman, Samia Ahmad

**Affiliations:** 1 Department of Radiology, University of Pretoria, South Africa; 2 Steve Biko Academic Hospital, Pretoria, South Africa

## Abstract

**Background:**

Benign prostate hyperplasia (BPH) remains a common cause of lower urinary tract symptoms (LUTS) in ageing men in South Africa and can impact significantly on the quality of life (QOL) of these patients. The Urology Department at Steve Biko Academic Hospital (SBAH) can generally only offer men with LUTS the following treatment options: watchful waiting, medical treatment and surgical management. In men with symptomatic BPH, who are refractory to medical treatment, where anaesthesia is contra-indicated because of co-morbidities or transurethral resection of the prostate (TURP) is contra-indicated because of the prostate size, the Urology and Radiology departments at SBAH recently introduced prostate artery embolisation (PAE).

**Aim:**

To assess the outcome of PAE in 10 men with LUTS, secondary to BPH, by comparing their urinary symptoms, QOL and prostate volume before and 3 months after they underwent PAE in the Radiology Department at SBAH.

**Method:**

The review included the first 10 men who had undergone therapeutic PAE for symptomatic BPH from May 2016 to September 2016. The subjective symptomatic feedback was assessed according to the International Prostate Symptom Score (IPSS) and the Global Quality of Life questionnaire, created by the American Urological Association (AUA). The reduction in the size of the prostate was measured on magnetic resonance imaging (MRI).

**Results:**

Embolisation was technically achieved in all 10 patients. Bilateral embolisation was performed on nine patients. One patient received unilateral embolisation secondary to unilateral tortuous and atherosclerotic changes of the iliac arteries. Within the 3-month follow-up, the mean IPSS score improved by 15.7 points (*p* < 0.0039), the mean QOL improved by 4.1 points (*p* < 0.0039) and the mean prostate volume reduction was 21.8 mL (*p* < 0.0039). Despite improvements observed, there was one clinical failure. No major complications were reported that increased hospital stay, required hospital readmission or required surgery.

**Conclusion:**

The study on the first 10 PAE performed in SBAH concludes that PAE is a safe and effective procedure with favourable short-term follow-up results. This indicates that PAE can safely be offered to patients, who are refractory to medical treatment and not suitable candidates for surgery, in urology departments such as in SBAH.

## Introduction

Benign prostatic hyperplasia (BPH) remains a common condition in ageing men, with a prevalence of 50% by the age of 60 years and 90% by the age of 85 years.^1^ Prostate gland enlargement may cause urethral compression and mechanical bladder outlet obstruction, leading to lower urinary tract symptoms (LUTS). While LUTS secondary to BPH is not life-threatening, it significantly alters patients’ quality of life (QOL) by interfering with their daily activities and sleep patterns.^1,2,3,4^

The International Prostate Symptom Score (IPSS), which was updated by the American Urological Association (AUA) in 2003, is a standard, valid questionnaire widely used to assess LUTS.^4^ The IPSS score rates patients’ subjective experiences of their urinary symptoms. The score can be used to identify, quantify and monitor LUTS, guiding the treatment indication. The score includes eight separate questions; seven symptom questions are scored on a scale of 0–5 points, 0 being not at all and 5 being almost always. The total score ranges from 0 to 35 points. The symptom categories include the following: mild (0–7 points), moderate (8–19 points) and severe (20–35 points). The eighth question assesses the global QOL, with men rating their feelings, should they have to live with their LUTS indefinitely, on a scale of 0 to 6 points, where 0 is delighted and 6 is terrible.^2,4,5,6,7^

In the past decade, the management of BPH was drastically modified by including minimally invasive treatment modalities. The current management options include the following: watchful waiting in patients with mild symptoms of LUTS and those who do not have bladder outlet obstruction, and medical and surgical management for patients with moderate or severe LUTS. Medical therapies include alpha-adrenergic receptor antagonists, 5-alpha-reductase inhibitors and anticholinergic agents. Minimally invasive therapies include visual laser ablation of the prostate, transurethral needle ablation, high-intensity focused ultrasound and transurethral microwave thermotherapy. Surgical procedures include transurethral incision of the prostate (TUIP) in prostates < 30 g, transurethral resection of the prostate (TURP) in prostates < 80–100 g and open prostatectomy in prostates > 80–100 g. Anaesthesia-free procedures include prostatic stent and prostatic urethral lift.^2,3,4,6,7,8^

Prostate artery embolisation (PAE) is a minimally invasive and alternative treatment option for symptomatic BPH. The first PAE was reported in 2000 in a patient with refractory haematuria and LUTS. Prostate artery embolisation was performed in treating the recurrent haematuria, with subsequent reduction in LUTS and prostatic volume.^9^

Steve Biko Academic Hospital (SBAH) in Gauteng province, South Africa, is a state-funded public-sector tertiary hospital which operates within tight financial constraints. These constraints mean that the Urology Department at SBAH is only able to offer the following: watchful waiting, alpha-adrenergic receptor antagonists or surgery to symptomatic men with BPH. TURP remains the standard surgical treatment for symptomatic BPH, while open prostatectomy is necessary for men with prostates larger than 80 g. Prostate artery embolisation is a relatively new, less invasive procedure offered to men in the Urology Department at SBAH, who are refractory to medical treatment and on the waiting list for prostate surgery, provided that there are no contra-indications.

No studies on the outcome of PAE for the treatment of LUTS secondary to BPH were published in South Africa at the time of this study. This assessment on the outcome of the first PAE performed at SBAH should be of great benefit to the Urology and Radiology departments at SBAH, continuing to improve and offer this relatively new procedure.

## Methods

The study was a retrospective record review of 10 men who had undergone PAE for symptomatic BPH at SBAH in 2016. The aim of this retrospective review was to assess the outcome of PAE for the treatment of LUTS, secondary to BPH, in the first 10 patients treated in the Radiology Department of SBAH in 2016.

**FIGURE 1:**
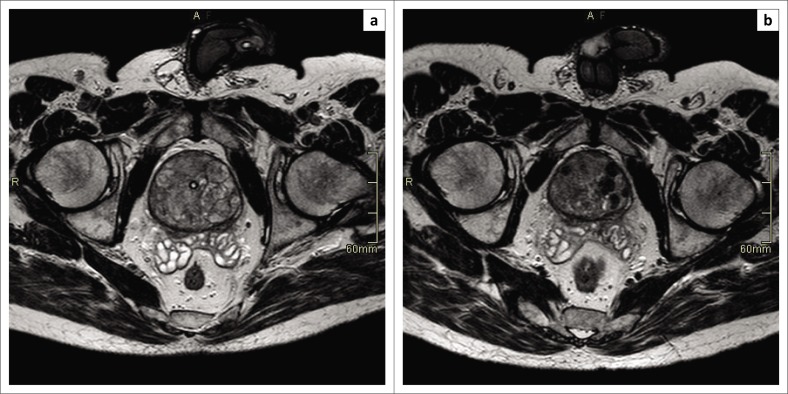
Magnetic resonance imaging of the prostate before and after prostate artery embolisation: (a) Magnetic resonance imaging before prostate artery embolisation with a transurethral catheter in situ. (b) Magnetic resonance imaging 3 months after prostate artery embolisation with the transurethral catheter removed and low-signal intensities in both prostate lobes because of ischaemia.

The objectives of the study were to determine whether there was any change in LUTS as assessed by pre- and post-IPSS questionnaire in 10 patients 3 months after therapeutic PAE for BPH, to determine whether there was any change in QOL as assessed by pre- and post-QOL questionnaire in 10 patients 3 months after therapeutic PAE for BPH, and to determine whether there was any change in prostate volume as measured by magnetic resonance imaging (MRI) in 10 patients 3 months after therapeutic PAE for BPH (Figure 1).

### Patient selection

This study was conducted on 10 men who underwent PAE for symptomatic BPH in the Radiology Department of SBAH in Pretoria, Gauteng province, South Africa. Ten men considered suitable for therapeutic PAE were selected by the staff in the Urology Department and then referred to the Radiology Department. The men selected had not responded to medical treatment, were not considered fit for general anaesthesia or were awaiting prostate surgery. No other treatment options were available to them at SBAH. The sample was thus a convenient sample consisting of the first 10 men who had undergone PAE for LUTS, secondary to BPH, in the Radiology Department at SBAH.

### The prostate artery embolisation procedure

**FIGURE 2:**
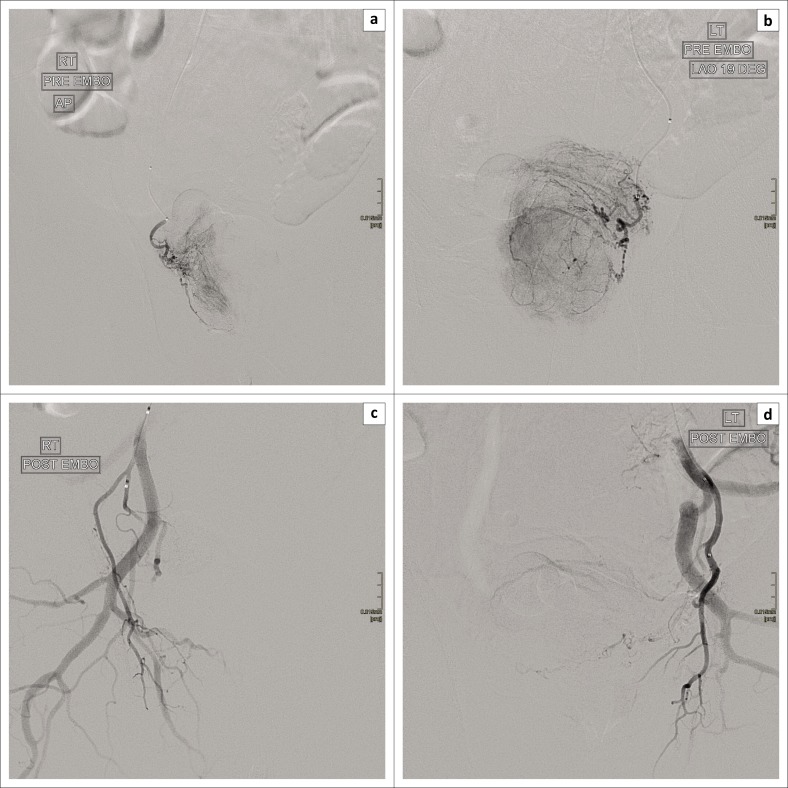
(a) Pre-embolisation digital subtraction angiography in the right anterior oblique projection with a prostatic blush. (b) Pre-embolisation digital subtraction angiography in the left anterior oblique projection with a prostatic blush. (c, d) Post-embolisation digital subtraction angiography with stasis of the prostatic arteries and absent prostatic blush.

The PAE procedures were all performed in the interventional theatre of the Radiology Department of SBAH. The men were admitted to hospital the day before PAE. A urinary catheter was inserted prior to the procedure, provided that there was no chronic urinary catheter in place. The procedure was performed under local anaesthesia with sedation in patients where general anaesthesia was contra-indicated because of co-morbidities and under general anaesthesia where there were no contra-indications. The radial or femoral artery was accessed using the Seldinger’s technique. A 5Fr sheath was used to facilitate catheter exchanges. A micro-catheter was used to super-select the prostatic arteries. Any collateral circulation to the bladder, rectum or penis was coil-embolised to avoid non-target embolisation. Embolisation with non-absorbable polyvinyl alcohol particles, under imaging guidance, was performed to an end point near-stasis once the micro-catheter was in the optimal position. This was confirmed with fluoroscopic imaging. The same technique was used to perform embolisation on the contra-lateral side for bilateral PAE (Figure 2).

The men with chronic urinary catheters were discharged with the catheter, which was to be removed at the 7–10-day follow-up, attempting to achieve spontaneous voiding. In the case where spontaneous voiding failed, spontaneous voiding was attempted weekly.

### Measurements

The patients’ subjective symptomatic feedback on the therapeutic outcome of PAE was determined by the administration of the AUA symptom score questionnaire pre-PAE and 3-months post-PAE by doctors in the urology clinic. The questionnaire included seven urinary symptom-related questions and one global QOL question created by the AUA in 1992.

Patients’ pre-PAE prostate volume was measured on the Philips Achieva 1.5T MRI in the 3 months before they underwent PAE and their post-PAE prostate volume was measured 3–4 months after the procedure. Prostate volume was calculated from the MRI measurements using the ellipsoid formula: π/6 × (transverse diameter × anteroposterior diameter × cephalocaudal diameter).

### Data analysis

The biographical and clinical records of 10 patients with LUTS, believed to be secondary to BPH, who had undergone therapeutic PAE from May 2016 to September 2016, were recovered from the SBAH patient record archive. Patients’ radiological images were recovered from the SBAH Picture Archiving and Communication System (PACS).

The patients’ biographical and clinical data were recorded including their age, gender, hospital number and contact details, as well as the dates of the PAE procedure and the pre- and post-PAE MRIs, and any major complications that they experienced. These data were entered into a standard statistical analytic programme where various descriptive statistical measures (mean, median, range and standard deviation) for pre- and post-PAE (prostate volume, IPSS and QOL) were calculated. As the distributions of the pre- and post-variables were not similar, a two-tailed Sign test was used to test the null hypothesis in preference to the Wilcoxon matched-pairs signed-rank test. The *p*-value was tested at the 5% significance level.

## Ethical consideration

Ethical approval to conduct the study was obtained from the Faculty of Health Sciences Research Ethics Committee of the University of Pretoria (Ethics reference number: 11/2017). Permission to use patient information was obtained from the SBAH Chief Executive Officer. Written, and where necessary verbal, informed consent was then obtained from the participants to use their anonymised data for medical research purposes.

## Results

Prostate artery embolisation was technically achieved in all 10 patients. Bilateral embolisation was performed on nine patients (90%). One patient received unilateral embolisation, secondary to unilateral tortuous and atherosclerotic changes of the iliac arteries.

The 10 patients were discharged the morning following the embolisation. Four of the 10 patients with chronic urinary catheters, prior to PAE, were discharged with a urinary catheter. The remaining six patients (without chronic urinary catheters before the PAE) were discharged without a urinary catheter, of which none presented with acute urinary retention.

Three of the four patients with chronic urinary catheters voided spontaneously after urinary catheter removal at the 1-week follow-up. The three patients were still catheter free at the 3-month follow-up. One patient with a chronic urinary catheter did not void spontaneously after multiple interval follow-ups and attempts. This patient is not catheter free after the PAE, indicating clinical failure.

Descriptive data analysis is described in Table 1 with variables including the prostate volume before and after PAE, the IPSS before and after PAE and the QOL before and after PAE.

**TABLE 1:** Response of variables before and after prostate artery embolisation.

Variables	Pre-prostate volume (mL)	Post-prostate volume (mL)	Pre-IPSS	Post-IPSS	Pre-QOL	Post-QOL
Minimum	12.4	8.6	15	3	4	0
Maximum	141.2	132.7	32	23	6	6
Mean	74.96	53.17	25.1	9.444444	5.7	1.6
Standard deviation	40.51793	36.04725	4.581363	7.108289	O.6749486	1.837873

IPSS, International Prostate Symptom Score; QOL, quality of life.

**FIGURE 3:**
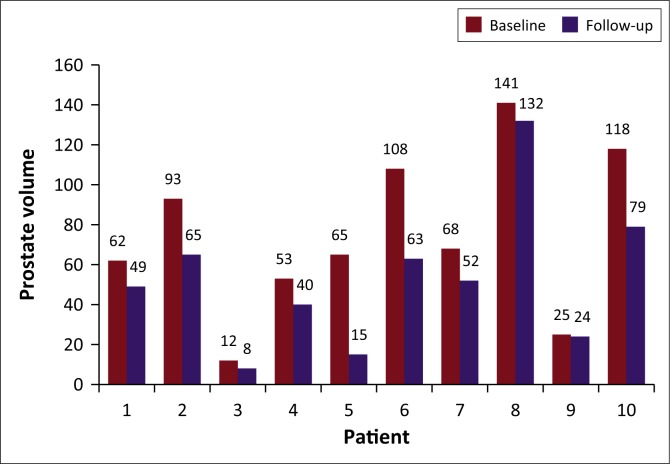
Prostate volume before and after prostate artery embolisation.

The prostate volume in nine patients as measured on MRI showed a mean reduction of 29%, ranging from 4% to 77% at the 3-month follow-up. The mean pre- and post-prostate volume was statistically significant, with a *p*-value of 0.0039 (*p* < 0.05) at a 5% significance level. Patient 4 was excluded from the mean prostatic volume analysis, although he showed a reduction in the prostatic volume as measured on ultrasound, as the MRI was contra-indicated because of a pacemaker. The prostate volume decreased in all 10 patients as outlined in Figure 3.

**FIGURE 4:**
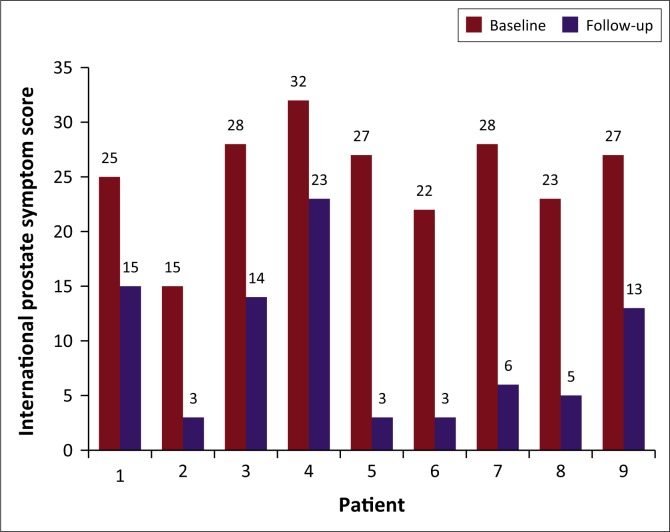
International Prostate Symptom Score before and after prostate artery embolisation.

The IPSS in nine patients showed a mean reduction of 62%, ranging from 0% to 89% at the 3-month follow-up. The mean pre- and post-IPSS was statistically significant, with a *p*-value of 0.0039 (*p* < 0.05) at the 5% significance level. Patient 10 was excluded from the mean IPSS analysis as he could not void spontaneously at all and the post-PAE AUA symptoms could not be assessed with the chronic urinary catheter in situ. The IPSS improved in 9 of the 10 patients as outlined in Figure 4.

**FIGURE 5:**
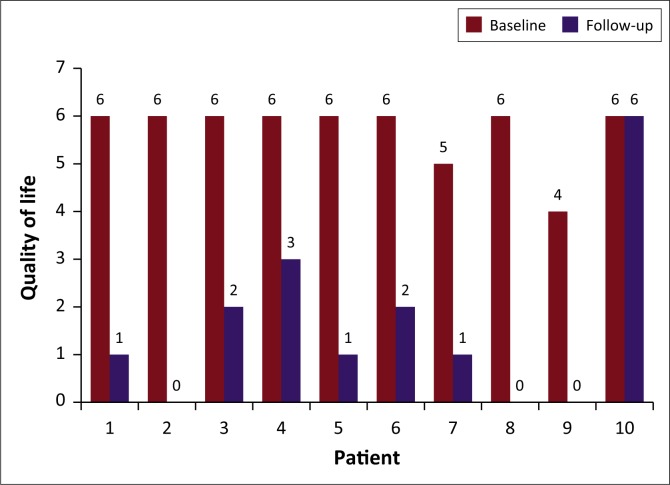
Quality of life before and after prostate artery embolisation.

The QOL in 10 patients showed a mean reduction of 72%, ranging from 0% to 100% at the 3-month follow-up. The mean pre- and post-QOL was statistically significant, with a *p*-value of 0.0039 (*p* < 0.05) at the 5% significance level. The QOL improved in all but one patient (Patient 10) who could not void spontaneously without a urinary catheter 3 months post-PAE, as outlined in Figure 5.

## Discussion

Since De Merrit et al.’s reporting of the first case of PAE in 2000, several further studies have shown the results of PAE for the treatment of symptomatic BPH.^9^ Short- and midterm outcomes after PAE in 255 patients were assessed by Pisco et al., indicating a 98% success rate.^10^ The early results from Bagla et al.’s clinical trial indicated that PAE was a safe effective treatment option for men with BPH.^11^ In Bagla et al.’s study, PAE was technically successful in 18 of 20 men (90%). Grosso et al.’s study on 13 men with BPH indicated that PAE was feasible, safe and efficacious in managing men with LUTS related to BPH.^12^ These studies of PAE for symptomatic BPH have shown PAE to be an effective therapeutic choice. In most studies, PAE was performed for the treatment of symptomatic BPH, and men with a bleeding diathesis, renal insufficiency, prostate cancer, bladder cancer, acute urinary retention or urethral stricture were excluded from these studies.^11,12,13^

Arterial catheterisation for PAE can be technically difficult in men with atherosclerotic iliac arteries or with anatomical variation in the origin of the prostate arteries.^10,11,12,13^ The complications of PAE can be categorised as minor self-limiting episodes, resolving spontaneously, and include pelvic pain, urinary frequency, haematuria, dysuria and haematospermia.^14^ Non-target embolisation is a major complication and includes risks such as bladder wall ischaemia, transient ischaemic rectitis and erectile dysfunction. Of these, the only major complication reported is bladder wall ischaemia by Pisco et al.^15^

The clinical outcomes for the first 10 men who underwent PAE at SBAH indicated a statistically significant improvement in IPSS, QOL and prostate volume over 3 months. No patients experienced periprocedural pain and no major complications were reported that increased hospital stay, required hospital readmission or required surgery. Despite these encouraging results, one patient unfortunately did not improve, which is recorded as a clinical failure. The findings of this study are consistent with the findings of other studies.^10,11,12^ The results are promising, despite the small number of men treated and the short follow-up period. Interestingly, one man who underwent unilateral embolisation had an improvement of all evaluated parameters over the 3 months.

The study has some limitations. Although IPSS is a widely used validated questionnaire, interviewer-administered questionnaires conducted with men may introduce an element of bias. The baseline AUA symptom score in the patients with chronic urinary catheters was recorded as the symptoms before the catheter was inserted and the patient may not remember the exact symptoms if the catheter had been in place for a long period. In this study, only a pre-PAE pelvic MRI was performed. Pre-PAE pelvic computerised tomography (CT) angiography for pre-procedural assessment of the anatomy and atherosclerotic changes of the iliac and prostatic arteries would also have been beneficial to predict intra-procedural difficulties and thereby decrease theatre and radiation time. A cone beam CT for intra-procedural localisation and assessment for procedural end-points is not available in the interventional theatre in SBAH, which may be beneficial. This study was limited to a small group of men studied for 3 months post-PAE. A longer follow-up period would allow the researcher to reach more definitive conclusions about the outcomes of this procedure in these men. No comparison was made between PAE and the medical or surgical management of BPH in this study.

The encouraging results of this study of the first 10 men treated for BPH by PAE at SBAH suggest that the Urology Department should continue to offer this less invasive intervention in centres with the appropriate skill and clinical support.

## Conclusion

The study on the first 10 PAE performed in SBAH concludes that PAE is a safe and effective procedure with favourable short-term follow-up results. This indicates that PAE can safely be offered to patients who are refractory to medical treatment and not suitable candidates for surgery. Larger case series, longer follow-up periods and comparative studies are required to further evaluate the role of PAE in SBAH.
